# Effects of the Blob on settlement of spotted sand bass, *Paralabrax maculatofasciatus*, to Mission Bay, San Diego, CA

**DOI:** 10.1371/journal.pone.0188449

**Published:** 2017-11-27

**Authors:** Anthony Basilio, Steven Searcy, Andrew R. Thompson

**Affiliations:** 1 Environmental and Ocean Sciences, University of San Diego, San Diego, California, United States of America; 2 Fisheries Resources Division, Southwest Fisheries Science Center, NOAA Fisheries Service, La Jolla, California, United States of America; Department of Agriculture and Water Resources, AUSTRALIA

## Abstract

The West Coast of the United States experienced variable and sometimes highly unusual oceanographic conditions between 2012 and 2015. In particular, a warm mass of surface water known as the Pacific Warm Anomaly (popularly as “The Blob”) impinged on southern California in 2014, and warm-water conditions remained during the 2015 El Niño. We examine how this oceanographic variability affected delivery and individual characteristics of larval spotted sand bass (*Paralabrax maculatofasciatus*) to an estuarine nursery habitat in southern California. To quantify *P*. *maculatofasciatus* settlement patterns, three larval collectors were installed near the mouth of Mission Bay, San Diego CA, and retrieved weekly from June–October of 2012–2015. During ‘Blob‘ conditions in 2014 and 2015, lower settlement rates of spotted sand bass were associated with higher sea surface temperature and lower wind speed, chlorophyll *a* (chl *a*) and upwelling. Overall, the number of settlers per day peaked at intermediate chl *a* values across weeks. Individual characteristics of larvae that settled in 2014–2015 were consistent with a poor feeding environment. Although settlers were longer in length in 2014–15, fish in these years had slower larval otolith growth, a longer larval duration, and a trend towards lower condition, traits that are often associated with lower survival and recruitment. This study suggests that future settlement and recruitment of *P*. *maculatofasciatus* and other fishes with similar life histories may be adversely affected in southern California if ocean temperatures continue to rise in the face of climate change.

## Introduction

A central goal of marine ecology and fisheries biology is to understand factors that contribute to variability in population size [1]. For many marine organisms understanding causes of fluctuations in abundance is complicated by a two-part life history with pelagic larvae that develop offshore and settle to benthic juvenile/adult habitats [2–5]. The number of larvae that reach settlement is a critical factor contributing to future year-class strength [3, 6–8], although post-settlement processes can also be an important source of variation [9, 10].

Settlement success is dependent on a variety of processes including egg production [11, 12] physical transport and retention of larvae [6, 13–18], and larval mortality [19]. Even small variations in larval mortality can lead to large changes in the number of individuals that survive this period [20]. Larval mortality may be caused directly by predation [21], as well as indirectly through environmental conditions such as water temperature and food supply [22–27]. Food availability is particularly important to larval survival because larvae have high metabolic rates and low energy reserves [28]. At elevated water temperatures, metabolic rates are faster, thereby increasing energetic requirements and increasing risk of starvation [22, 23, 25, 29, 30]. After only a few days without food, the larvae of some fish species reach a "point of no return" and are unable to survive [31–33].

Water temperature and food availability may also affect larval survival by influencing growth rates and physiological condition [34–37]. The growth mortality hypothesis [38] suggests that if mortality is lower for larger individuals, then faster growing individuals of a given age will have a lower probability of mortality than slower growing individuals of the same age [39]. Larger individuals may be able to detect and respond to predators more effectively [21, 40], obtain food and withstand starvation [21, 41, 42], although, some studies suggest that larger larvae may actually have higher mortality [39, 43–45]. Finally, larvae with faster growth may be exposed to overall lower levels of predation by quickly growing out of this vulnerable life history stage [38, 46, 47].

Increased sea surface temperature (SST), may also be related to poor larval growth conditions by indicating periods with low upwelling and potential stratification of the water column [48, 49]. During upwelling events, increased supply of nutrients to the surface water can spur phytoplankton growth and support a trophic pyramid that includes zooplankton and many fish species [50]. When upwelling ceases, SST and stratification increase, and there is decreased productivity [48]. Indeed, previous work indicates that during a long-term shift from a cool to a warm water regime as the result of changing Pacific Decadal Oscillation there was an overall 46% reduction in volume of phyto- and zooplankton and a corresponding decline in abundance of many species of larval rockfish (*Sebastes sp*.) [51].

Understanding how settlement is affected by environmental conditions such as water temperature is especially important to gain insight into how warming ocean conditions may impact marine populations. In the northeast Pacific Ocean, during the winter of 2013–14, weakened winds and unusually high sea level pressure formed a region where heat was retained in surface water. This mass of warm water, known as “the Blob”, reached coastal waters in the western United States in the spring/summer of 2014 [52] and resulted in widespread changes in the biological structure and composition of both open-ocean and coastal ecosystems [53]. Sea surface temperatures were 1–4°C higher than average along the west coast of North America [53]. A geographically distinct section of the Blob near San Diego was named the Southern California Warm Anomaly (SCWA) and increased thermal stratification which led to a reduction of vertical mixing and nutrient fluxes into the surface water [54]. This pool of warm water was partially responsible for the shift in the California Current from a productive La Niña state in 2013 to a warm area with low productivity [55].

Our objective was to examine how changes in environmental conditions over four years (2012–2015) affected daily and annual settlement success of spotted sand bass (*Paralabrax maculatofasciatus*) in Mission Bay, CA. We also examined how environmental conditions influenced individual characteristics at settlement (length, age, condition, larval growth rates). The sampling years included two years of relatively normal conditions (2012–13) followed by the arrival of the SCWA in 2014 and dramatic changes in chlorophyll a (chl *a*) and SST that continued through 2015.

## Materials and methods

### Ethics statement

Sampling was conducted under California Department of Fish and Wildlife scientific sampling permit SC-11846 with the approval of the University of San Diego’s Institutional Animal Care and Use Committee (IACUC).

### Study species

Spotted sand bass (*Paralabrax maculatofasciatus*) are a recreationally important fish in Southern California typically found from Santa Monica Bay, CA in the north to Mazatlan, Mexico in the south [56]. There are two main populations of *P*. *maculatofasciatus* along this range: one in the Gulf of California and another in the Pacific along Baja California and Southern California [57]. There is likely a high degree of larval retention as *P*. *maculatofasciatus* sampled near San Diego are genetically distinct from those further south along the Baja California peninsula [57]. Spotted sand bass adults are primarily found in calm, shallow, nearshore habitats such as bays and estuaries [56]. Adults spawn near the entrance of bays in the late spring and summer [56], are capable of spawning multiple times throughout the season and may even spawn daily [58]. Larvae develop in the coastal ocean for approximately 28 days [59] after which they return to settle in bays and estuaries that contain structured habitats such as eelgrass, surfgrass, and rock relief [60].

### Environmental data

We measured several environmental parameters that were hypothesized to affect settlement dynamics including daily SST and chl *a*. Phytoplankton, as represented by chl *a* concentration, are consumed by zooplankton and are often used as a proxy for food availability for larval fishes [61, 62]. Significant positive relationships between chl *a* concentration and abundance of copepods have been reported in a diverse range of environments such as offshore temperate [63, 64] and tropical waters [65, 66], and in shallow estuaries [67–69].

Chl *a* concentration and SST, measured between May and October, were obtained using the R package Xtractomatic [70], which downloaded satellite measurements for SST (Pathfinder v.5, 5.5 km resolution, 8 day composite) and chl *a* (MODIS Aqua, 2.5 km resolution, 8 day composite) from NOAA’s CoastWatch Browser website (http://coastwatch.pfeg.noaa.gov). Both variables were estimated for an offshore region (34°25’24” N and 122°06’05”W to 29°56’23”N and 115°49’38”W) that encompassed the potential area that larvae arriving to San Diego might be spawned from [57], based on historical offshore distribution [71], as well as potential transport of larvae given a larval duration of 28 days [59] and an average net alongshore transport rate of 0.2 m/sec [72].

Mean wind speed data were obtained from San Diego International Airport provided by the Automated Surface Observation System from Weather Underground [73]. These measurements were based on 24 separate hourly measurements made throughout each day. Upwelling index was accessed from the NOAA Pacific Fisheries Environmental Laboratory database, station 93950–2097, which collects measurements of atmospheric pressure at mean sea level every six hours.

### Larval fish collection and morphometric analysis

**Fig 1:**
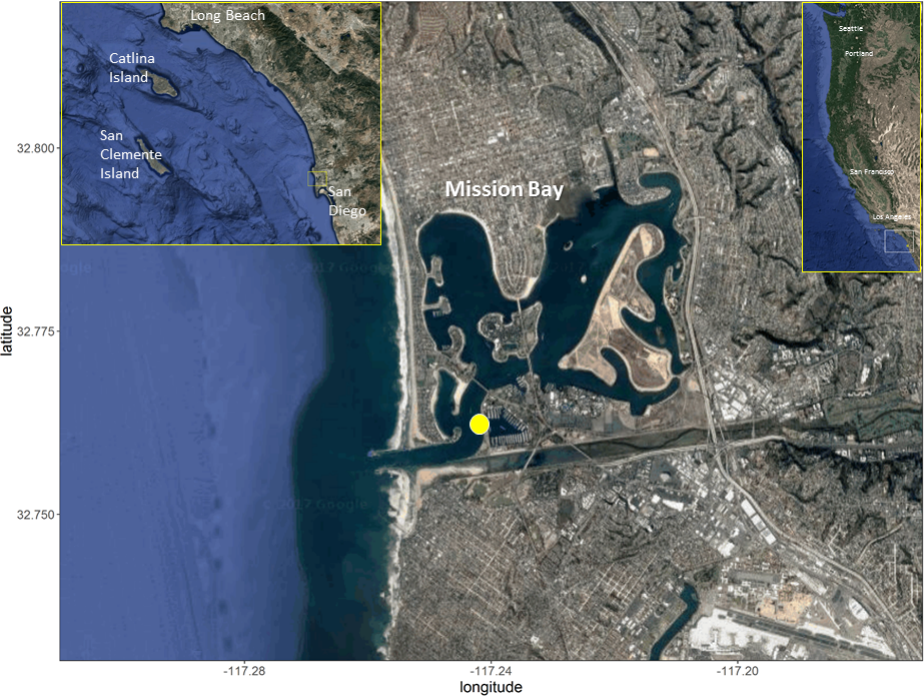
Mission Bay, San Diego, CA. Map of Mission Bay (main map), southern California (left inset), and the west coast of North America (right inset). Yellow circle within main map demarks location of fish collectors. Maps produced using R code [76].

Recently settled *P*. *maculatofasciatus* were collected weekly from June–October in 2012, 2013, 2014 and 2015 near the Mission Bay inlet, San Diego, CA (Fig 1). Fish were collected using a modification of the standard monitoring units for the recruitment of fishes (SMURFs) design [74]. Each collector (0.5 x 0.18 m dia.) consisted of a narrow cylinder of plastic fencing material with a 2.5 cm grid filled with giant kelp (*Macrocystis pyrifera*). The kelp attracts juvenile fish that use this habitat as a shelter, and the fencing material prevents access by larger predators [75]. Three replicate collectors were positioned 5 m apart in approximately 4 m of water and attached to a cinder block anchor with a brass clip and floated upright with a Styrofoam float. Collectors were placed near the bottom to ensure they would not be disturbed by boat traffic, and were the only structure along the sandy bottom of the surrounding area thereby maximizing the chance that late stage larvae arriving to this area would shelter in them. Collectors were retrieved by snorkeling to depth, enclosing the collector in a 1 mm mesh bag, unclipping it and bringing it back to shore where all fishes were removed, euthanized with hypothermic shock and preserved in 70% ethanol for later identification. Following collection, the kelp was investigated and if it had started to deteriorate, the old kelp inside the collector was replaced and the collector was returned to its mooring.

In the laboratory, standard length of each fish was measured from individual digital images using Image-J analysis software [77]. Images were obtained by placing each fish on a calibrated slide and using a digital video camera attached to a dissecting microscope. Next, for age determination (see below), the sagittal otoliths were removed. Following otolith extraction, fish dry weight was obtained by placing fish into a drying oven at 60°C for 24 h, allowing them to cool for 5 min and weighing them on an analytical balance to the nearest 0.1 mg. Finally, body condition at settlement was estimated with the equation: condition = dry weight x standard length^-3^ [78].

### Otolith analysis

Sagittal otoliths were mounted on microscope slides using thermoplastic glue (Crystal Bond). One sagitta was randomly chosen and polished to the core using 200–600 grit sandpaper. All abnormally shaped and unclear (large portions with no discernable increments) otoliths were discarded (n = 9 from a total of 102). Images of sagitta were taken using a transmitted light microscope at 250x equipped with a digital camera. All otoliths were measured using an Image-Pro image analysis system [59]. Otoliths were measured along their longest radius from the core to the outer edge and distance from the core to each increment was measured and counted. Two days were added to all age estimates to account for the delay in daily growth ring formation in the larval otolith before yolk absorbtion [59]. Each sagitta was read blind, and after completing all sagittae once, they were read blind again. If counts differed by ≥ 3 increments (~10%) between the two readings, the otolith was reread. If the third count was within 1 count of the former readings, then one of the readings was randomly chosen for analysis. Alternatively, if the difference was > 1 of the previous readings, the otolith was discarded [79].

Fish collectors were only retrieved once a week, therefore records of daily settlement required us to back calculate settlement dates. Fortunately, *P*. *maculatofasciatus* have a ‘settlement check’ on their otoliths, identified as an abrupt change in the width of daily bands at the time of settlement [59]. This check is likely the result of stress or changes in growth or metabolic rate associated with the transition from a pelagic to a demersal life style [80]. If no check was apparent, the fish was considered to be collected during its initial process of settlement. From these measurements, larval duration and average pre-settlement otolith growth were determined. Finally, to estimate standard length at settlement for all fish collected, we calculated separate linear regressions for 2012, 2013, and 2014–2015 for the relationship between standard length at collection to age at collection and then used the equation of the line to back-calculate standard length at settlement [59]. Data for 2014–2015 were pooled due to low sample size in 2015 (n = 5).

### Data analysis

#### Annual scale

To determine whether there were significant differences in environmental conditions, average weekly settlement among years, and larval traits (age-, length-, condition-at-settlement), we first examined these factors for normality and homogeneity of variance. To test for significance among years, we used an ANOVA or the nonparametric counterpart (Kruskal-Wallis). When significant differences in these factors occurred among years, Tukey’s HSD means comparison test or the nonparametric equivalent (Wilcoxon rank sum test) were performed. Small sample sizes in 2014 and 2015 made it difficult to determine statistical differences in larval traits among years. Therefore, to estimate the minimum sample size required, post-hoc power analysis was run (alpha = 0.05).

All annual means of environmental variables were calculated by averaging conditions from May–October to incorporate the period from first potential spawning (approximately 1 month prior to our first sample) until the final retrieval of collectors.

#### Daily scale

To determine the importance of SST and chl *a* on settlement, we tested the plausibility of 6 *a priori* candidate models that included combinations of environmental parameters for all four years combined. We included both linear and quadratic terms for SST and chl *a* because we hypothesized that optimal settlement may occur at intermediate values. Because the settlement data were not normally distributed, we explored the fit of alternative distributions with generalized linear models (negative binomial, Poisson, zero-inflated) and determined that a negative binomial distribution with a log link best fit the data. Preliminary model exploration also indicated that it was not necessary to add year as a random effect under a mixed model platform. However, residuals from the negative binomial models were temporally autocorrelated at lags (days) 1–8, violating the assumption that each settlement sample was independent [81]. We thus utilized negative binomial models that accounted for serial temporal autocorrelation as implemented by the R package tscount [82]. Preliminary analyses revealed that the tscount models eliminated low-order residual temporal autocorrelation. Relative model plausibility was evaluated based on Akaike’s Information Criterion (AIC) scores (e. g., [83]). Overall model fit was determined by calculating the proportion of deviance explained by the most plausible model.

## Results

### Annual scale

**Fig 2:**
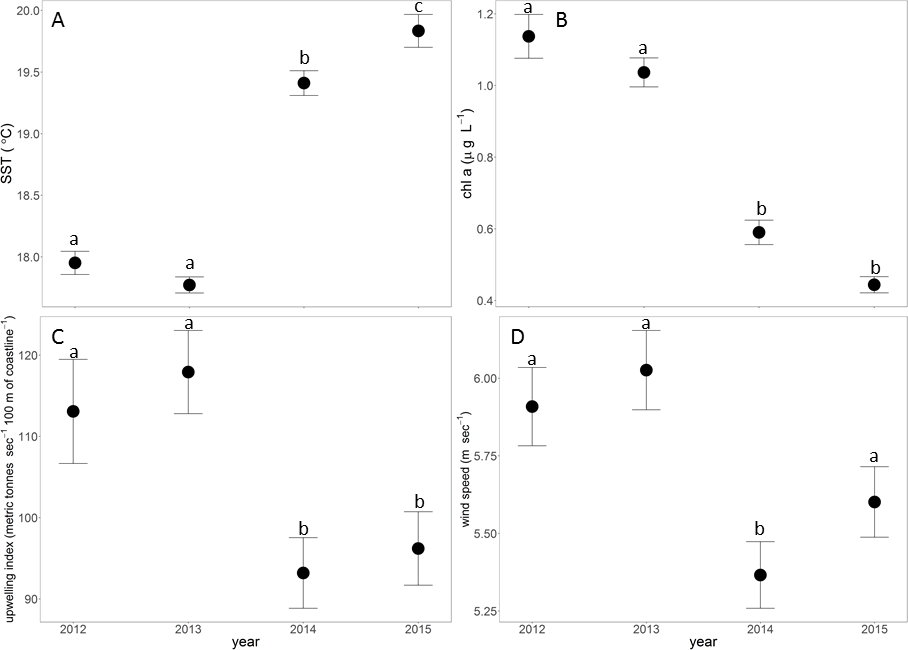
Oceanographic and wind conditions among years. A. Sea surface temperature, B. Chlorohpyll *a*, C. Upwelling index, D. Wind. Error bars are 1 standard error. Lower case letters above error bars depict significant pairwise comparisons among years.

Oceanographic and wind conditions were significantly different among years (Table 1). Annual mean SST was significantly lower in 2012 and 2013 relative to 2014 and 2015 (Fig 2A), whereas chl a, upwelling index and wind speed were significantly higher in 2012–13 compared to 2014–15 (Fig 2B–2D).

**Table 1:** Summary of Kruskal -Wallis results for inter-annual comparisons of environmental conditions from 2012, 2013, 2014, and 2015.

	χ2	df	p-value
Sea Surface Temperature (°C)	187.02	3	<0.0001
Chlorophyll *a* (μg L^-1^)	170.85	3	<0.0001
Wind Speed (m sec^-1^)	15.04	3	<0.001
Upwelling index	14.1	3	<0.001

**Fig 3:**
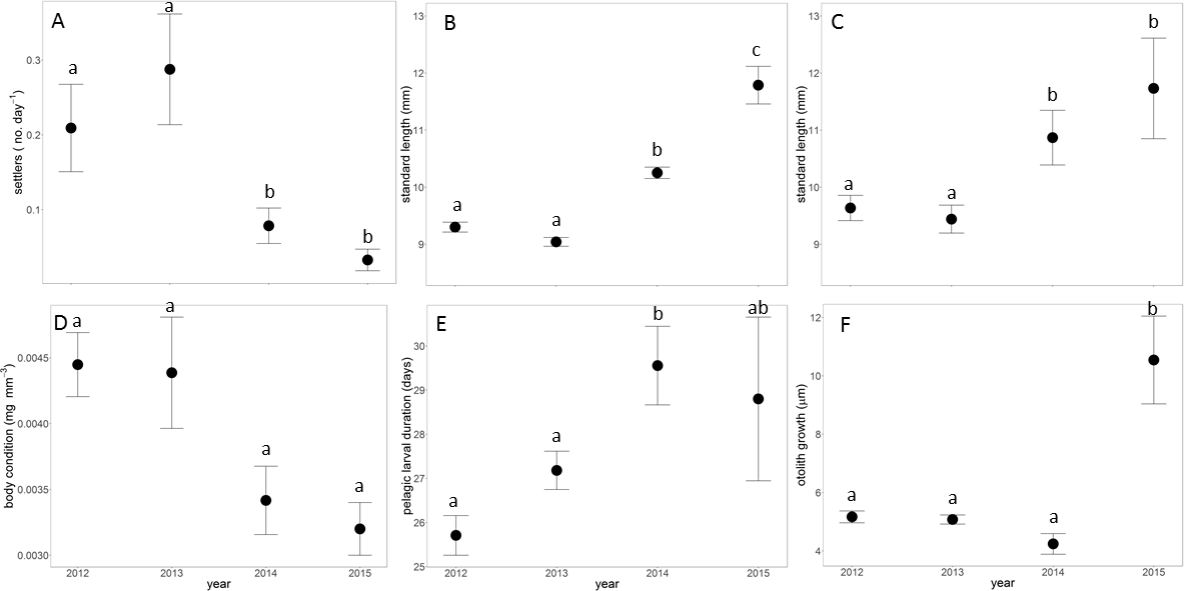
Annual trends in settlement and characteristics of settlers. A. Mean number of settlers, B. Size at settlement, C. Size at collection, D. Body condition, E. Pelagic larval duration, F. Otolith growth. A total of 32, 44, 12, and 5 fish were collected in 2012, 2013, 2014, and 2015, respectively. Error bars are 1 standard error. Lower case letters above error bars depict significant pairwise comparisons among years.

The abundance and individual characteristics of *P*. *maculatofasciatus* settlers also varied significantly among years (Table 2). Significantly more settlers were collected in 2012 and 2013 than 2014 and 2015 (Fig 3A). Individual characteristics also varied annually with mean standard length on the day of collection as well as back-calculated length at settlement lowest in 2012 and 2013 and increasing in 2014 and 2015 (Fig 3B and 3C). Body condition, a measure of body fatness, showed a trend of fish with higher condition in 2012 and 2013 than 2014 and 2015 (Fig 3D). Otolith derived traits also had mixed trends with fish in 2012 and 2013 settling at a younger average age than fish in 2014 and 2015 (Fig 3E). Due to the high degree of error associated with the small sample size in 2015 (n = 5), mean pelagic larval duration in that year overlapped with both 2013 and 2014. Finally, daily larval otolith growth had a non-significant trend to be lower in 2014 than in 2012 and 2013 with highest growth occurring in 2015 (Fig 3F). Overall, small sample sizes in 2014 and 2015 made it difficult to determine significant statistical differences in larval traits among years. Post-hoc power analysis suggested that the 2015 sample size (n = 5), assuming a moderate effect size (0.25) and a standard alpha (0.05), would result in relatively low power (0.12). With these same parameters, using the 2014 sample size (n = 12) power was still low (0.25). To obtain higher power (e.g. 0.6), sample size would need to be approximately n = 30 for all groups.

**Table 2:** Summary of inter-annual comparisons of individual larval characteristics for 2012, 2013, 2014, and 2015.

Measure	Test	F (ANOVA) or χ2 (Kruskal-Wallis)	df	p-value
Standard length at settlement	ANOVA	6.07	3	<0.0001	
Standard length at collection	ANOVA	5.56	3	<0.001	
Pelagic larval duration	ANOVA	6.07	3	<0.0001	
Larval otolith growth	Kruskal-Wallis	17.47	3	<0.001	
Body condition	Kruskal-Wallis	4.70	3	= 0.19	

### Daily scale

**Fig 4:**
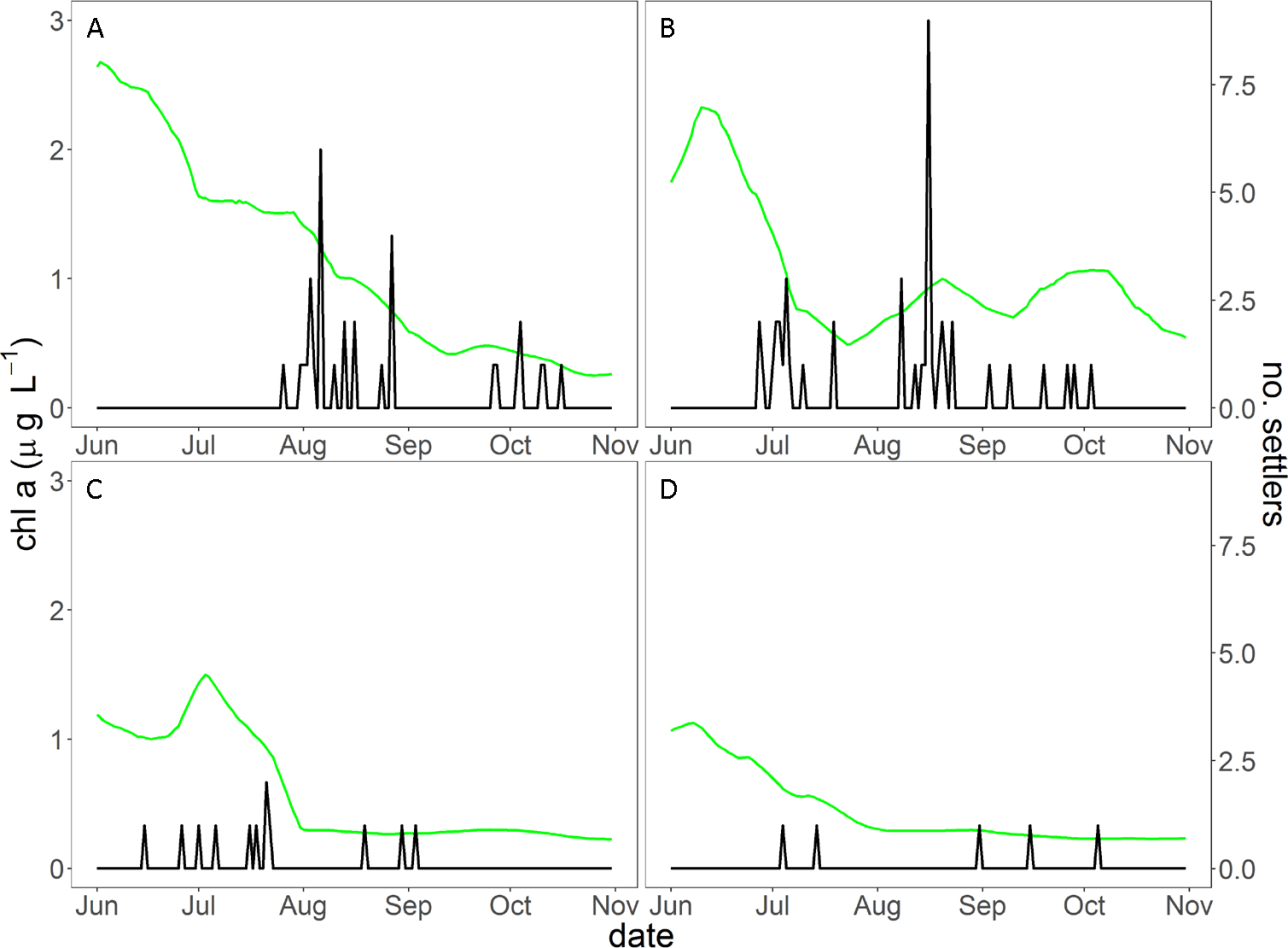
Relationship between chlorophyll *a* and number of settlers. Daily measurements of chlorophyll *a* (green line, left axis) and number of settlers in A. 2012, B. 2013, C. 2014, and D. 2015.

**Fig 5:**
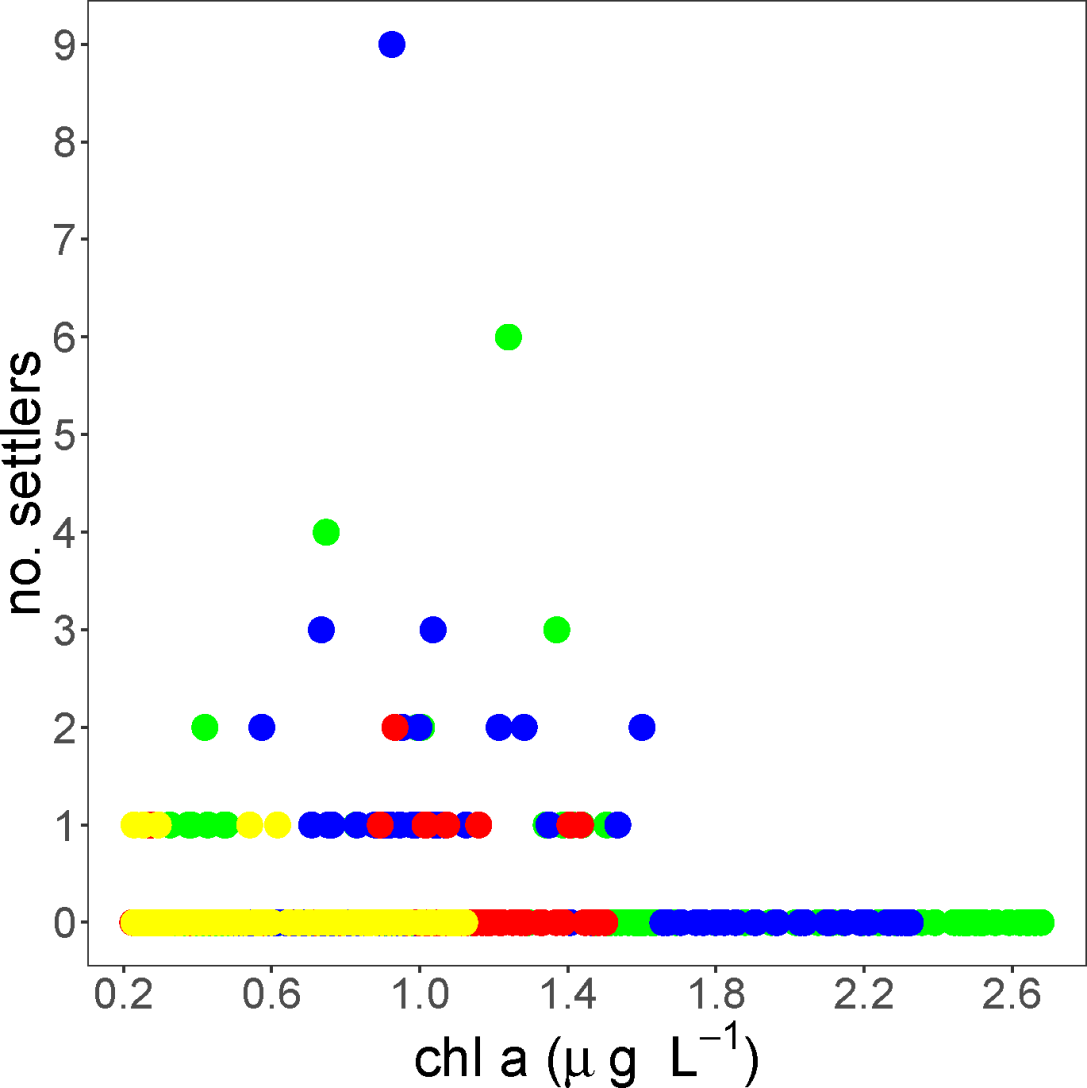
Scatterplot between chlorophyll *a* value and settlement for each day of the study. Green points are from 2012, blue from 2013, red from 2014, and yellow from 2015.

The only models that received support based on AIC scores contained (chl a)^2^, indicating that settlement was highest at intermediate values of chl a (Table 3). The best model, which had a weight of 79%, explained 22% of the total deviance. All terms in the best model had slope estimates that did not overlap 0 (Table 4). The second most plausible model contained sst + sst^2^ in addition to chl a + (chl a)^2^ and had a weight of 21%. Examination of slope estimates, however, revealed that 95% confidence intervals of slopes for both sst and sst^2^ overlapped 0 indicating that there was no effect of temperature on daily settlement abundances. Examination of time-series plots (Fig 4) and scatter plots of chl *a* and settlement (Fig 5) indicated that peak chl *a* was observed approximately two weeks prior to the onset of settlement and that peak settlement occurred at intermediate levels of chl *a*.

**Table 3:** Model selection results for parameters explaining variation in settlement.

model	AIC	delta AIC	k	model weight
chl a + (chl a)^2^	469.5	0.0	3	0.79
sst + sst^2^ + chl a + (chl a)^2^	472.2	2.7	5	0.21
sst + sst^2^	502.2	32.6	3	0
sst	503.2	33.6	2	0
sst + chl a	505.2	35.7	3	0
chl a	509.8	40.2	2	0

Delta AIC is the difference in AIC between a particular model and the most plausible model. k is the number of parameters in a model.

**Table 4:** Summary of results from the most plausible daily settlement model (chl a + (chl a)^2^).

	slope	se	lower 95% CI	upper 95% CI
intercept	-5.1	0.6	-6.3	-3.9
beta 1	0.9	0.4	0.1	1.6
chl a	7.0	1.4	4.2	9.8
chl a^2^	-3.2	0.7	-4.6	-1.7
sigma squared	2.9	NA	NA	NA

Beta 1 is the autocorrelation parameter and sigma squared the negative binomial overdispersion factor. se is standard error.

## Discussion

Settlement of spotted sand bass to Mission Bay was higher in 2012–13 than in 2014–15. We attribute the overall reduction in settlement in 2014 and 2015 to the arrival of the Blob (SCWA) in 2014 and continued warm conditions in association with El Niño in 2015. The Blob, which formed in the northeast Pacific in 2013, and then spread south and impinged upon southern California in May of 2014, brought with it unusually warm surface water and low chl *a* [49, 52, 84]. Low chlorophyll can likely be attributed to increased thermal stratification and reduction in nutrient fluxes from colder deep waters [53]. During the SCWA, compared to the previous 30 years, stratification was the strongest, surface nutrient levels of nitrate were approximately 0.02 μM lower, and chl *a* values were the lowest observed over the entire time period [49]. Similar patterns have been observed in previous work which suggests there is an inverse relationship between SST and productivity in the California Current Ecosystem (CCE) [85, 86], as well as more generally in the oceans [48].

In our study, low chl *a* levels were related to low settlement success, which supports the idea that larval food availability and larval survival are tightly linked [24, 87, 88]. Further, there was a delay between peak chl *a* and settlement. This likely reflects the time between the onset of primary production (phytoplankton) and development of secondary production (zooplankton) upon which larval fishes feed. Low offshore chl *a* levels, like those observed during the SCWA, are thought to indicate a poor feeding environment for larval fishes which consume small zooplankton such as copepods. Indeed, areas with high chl *a* have been related to enhanced recruitment of larvae that consume phytoplankton directly (e.g. barnacles) and indirectly (e.g. rockfish) across the entire CCE [62]. A recent study examining recruitment of a temperate wrasse, *Coris julis*, in the Azores archipelago found a strong relationship between ocean productivity and year-class strength at two different spatial scales and over a six year period [26]. One of the main differences between the current study and previous work is that in addition to annual trends, we were able to examine daily settlement pattern in relation to environmental conditions that each larva experienced. The shorter temporal scale allows for greater understanding of why settlement may vary over an entire recruitment season.

Although observed inter-annual variation in individual characteristics may be due to differences in selective mortality across years [41, 44, 45], we suggest that there is a close relationship between the larval feeding environment and individual larval characteristics. The idea that chl *a* reflects the feeding environment that larvae are exposed to is consistent with the individual characteristics of larvae that we measured. For example, settlers in 2014 and 2015, years characterized by low chl *a* levels, had a non-significant trend towards lower body condition which suggests that larvae may have had trouble finding sufficient food. In addition, in 2014 and 2015 larvae settled at an older age which may indicate that they needed to extend their larval duration, thereby increasing the time that larvae are vulnerable to high mortality [39, 89]. Interestingly, larval otolith growth rates in 2014 and 2015 were mixed, with lower otolith growth in 2014 and higher otolith growth in 2015. Lower otolith growth rates in 2014 are consistent with a poor feeding environment, whereas, higher otolith growth rates in 2015 may reflect warmer water temperatures or a decoupling between otolith growth and somatic growth [90].

The increased larval duration in 2014–15 may also be the result of changing current patterns with the introduction of the SCWA, which could prevent larvae from reaching the coast and delay settlement. A recent study in the CCE examined how movement of the warm anomaly to coastal waters in 2014 changed current patterns and altered settlement in 30 species of nudibranchs [91]. Northward ranges of typically southern nudibranch species may have been facilitated by increased poleward and onshore transport of their planktonic larvae [91]. This stronger poleward current is expected during periods of reduced upwelling, which was observed in 2014 off northern Baja California and southern California [55]. We expect a poleward shift in the CCE would not affect settlement of *P*. *maculatofasciatus* in Mission Bay, because Mission Bay is located near the northern end of their range. Any poleward movement alongshore would likely bring larvae to the bay from populations further south.

The lack of a strong link between SST and settlement does not preclude the idea that water temperature may indirectly affect larval survival by modifying plankton distribution and abundance, as well as by influencing larval metabolic rates and the amount of food required to survive [26]. Interestingly, previous studies have found a positive relationship between annual recruitment of spotted sand bass and mean summer SST off southern California [56, 92]. In particular, annual abundance was greater during times of higher SST, such as the El Niño events of 1977–78 and 1982–83 [56, 92]. The opposite trends between our study and the previous work may be due to the other studies surveying older fish, in contrast to our study which examined initial settlement of larvae, or may simply be due to factors associated with the SCWA that were not observed in previous years.

Another possible explanation for the decreased settlement in our study involves increased SST affecting reproduction. Although reproduction and recruitment rates are positively associated with SST in Pacific sardine (*Sardinops sagax*) [93], increased SST has been shown to negatively affect spawning success and egg production in demersal species in southern California such as the blackeyed goby (*Rhinogobiops nicholsii*) and garibaldi (*Hypsypops rubicundus*, [92]). *H*. *rubicundus* actually left their shallow nests within a harbor due to increased water temperature [92]. Although it is possible that *P*. *maculatofasciatus* may also abandon their spawning grounds for deeper areas as water temperatures rise, spotted sand bass are typically considered a warm water species. In San Diego, spotted sand bass are at the northern end of their range which extends into much warmer waters throughout the Gulf of California [56]. Furthermore, *P*. *maculatofasciatus* typically produces more eggs throughout the summer as water temperature increases, with the highest egg production occurring during July when the water is warmest [56, 94]. Also, unlike other members of *Paralabrax* common in southern California such as kelp bass (*P*. *clathratus*) and barred sand bass (*P*. *nebulifer*), spotted sand bass adults typically remain in shallow coastal embayments their entire lives and may be especially resistant to higher water temperatures [56].

We propose that in 2014–15 increased SST associated with the Blob and an El Niño, combined with decreased food availability due to nutrient stratification, formed an environment that limited survival of larval *P*. *maculatofasciatus*. The appearance of Blob conditions is highly unusual in the northeastern Pacific since detailed oceanographic monitoring began in the late 1940s. However, warming of surface waters is consistent with predicted ocean warming with climate change [95–97]. Although caution should be used to avoid over-interpreting our data due to the one study species and location that were examined, this study gives us insight into how increasing water temperatures and decreasing upwelling and chl *a* may affect settlement and recruitment patterns of fishes in southern California.

## Supporting information

S1 File Larval and environmental characteristics. Individual characteritsics of recently settled larvae and corresponding physical environmental variables.
(XLSX)
